# Butylated Hydroxytoluene (BHT) Protects SH-SY5Y Neuroblastoma Cells from Ferroptotic Cell Death: Insights from In Vitro and In Vivo Studies

**DOI:** 10.3390/antiox13020242

**Published:** 2024-02-17

**Authors:** Parisa Faraji, Astrid Borchert, Shahin Ahmadian, Hartmut Kuhn

**Affiliations:** 1 Department of Biochemistry, Charité—Universitätsmedizin Berlin, Corporate Member of Freie Universität Berlin and Humboldt Universität zu Berlin, Charitéplatz 1, 10117 Berlin, Germany; parisa.faraji@charite.de (P.F.); astrid.borchert@charite.de (A.B.); 2 Institute of Biochemistry and Biophysics, University of Tehran, Tehran 1417614335, Iran

**Keywords:** lipid peroxidation, biomembranes, iron metabolism, free radicals, Alzheimer’s disease, anti-oxidants, glutathione peroxidase

## Abstract

Ferroptosis is a special kind of programmed cell death that has been implicated in the pathogenesis of a large number of human diseases. It involves dysregulated intracellular iron metabolism and uncontrolled lipid peroxidation, which together initiate intracellular ferroptotic signalling pathways leading to cellular suicide. Pharmacological interference with ferroptotic signal transduction may prevent cell death, and thus patients suffering from ferroptosis-related diseases may benefit from such treatment. Butylated hydroxytoluene (BHT) is an effective anti-oxidant that is frequently used in oil chemistry and in cosmetics to prevent free-radical-mediated lipid peroxidation. Since it functions as a radical scavenger, it has previously been reported to interfere with ferroptotic signalling. Here, we show that BHT prevents RSL3- and ML162-induced ferroptotic cell death in cultured human neuroblastoma cells (SH-SY5Y) in a dose-dependent manner. It prevents the RSL3-induced oxidation of membrane lipids and normalises the RSL3-induced inhibition of the intracellular catalytic activity of glutathione peroxidase 4. The systemic application of BHT in a rat Alzheimer’s disease model prevented the upregulation of the expression of ferroptosis-related genes. Taken together, these data indicate that BHT interferes with ferroptotic signalling in cultured neuroblastoma cells and may prevent ferroptotic cell death in an animal Alzheimer’s disease model.

## 1. Background

Ferroptosis is a special form of regulated cell death that is characterised by the excessive intracellular deposition of iron ions and strongly upregulated lipid peroxidation, which exceeds the anti-oxidative capacity of the cells [1,2,3]. It was first discovered some 10 years ago [4] as a programmed cell-death pathway, which is initiated under certain metabolic conditions in tumour cells to prevent excessive cell proliferation and thus tumour growth. Although intracellular ferroptotic signalling can be initiated in different ways and although a number of different ferroptotic signalling cascades have been described [5,6,7], the final cell-death mechanisms are rather simple. Ferroptotic cell death involves three major metabolic hallmarks, which partly interfere with each other: (i) Intracellular iron deposition [8]: These iron deposits are a functional consequence of a dysregulated cellular iron homeostasis that usually develops when the transferrin receptor-mediated iron import exceeds the cellular iron export. Iron is essential for the function of all cells but excessive intracellular iron accumulation is clearly detrimental [9,10]. (ii) Reduced anti-oxidative capacity: Under normal conditions, the intracellular redox homeostasis is usually balanced and there is an equilibrium between pro- and anti-oxidative reactions [11,12]. When pro-oxidative reactions are upregulated or when anti-oxidative processes are downregulated, oxidative stress is induced and this alters the functional phenotype of the cells [13]. In ferroptosis, the anti-oxidative defence system is frequently impaired, which is either caused by the downregulation of glutathione peroxidase 4 (GPX4) expression, by the inhibition of its catalytic activity [14], or by the dysfunction of the NAD(P)H/ferroptosis-suppressor protein-1/ubiquinone axis [15]. (iii) Elevated lipid peroxidation: Since ferrous iron (Fe^2+^) is a strong oxidant, intracellular iron accumulation increases the cellular oxidation potential [9,10]. Under aerobic conditions, ferrous iron (Fe^2+^) delivers an electron to molecular dioxygen, which leads to the formation of ferric iron and superoxide (•O_2_·^−^). This reactive oxygen species (ROS) can then initiate free radical-mediated secondary reactions, such as the peroxidation of unsaturated membrane lipids [16].

Butylated hydroxytoluene (BHT, 2,6-di-*tert*-butyl-4-methylphenol) is a lipophilic anti-oxidant [17]. Since it prevents free radical-mediated oxidation reactions [17,18], it has previously been used as a preservative in food chemistry. Although it exhibits anti-viral properties [19], it was not approved by the FDA for anti-viral therapy. As with many related phenolic anti-oxidants, BHT has low acute toxicity [20], and the overall evaluation of the potential health risks of BHT application was rather positive [21,22]. On the other hand, several studies suggested a possible link between BHT intake and the risk of developing cancer [23,24,25], and because of this uncertainty the routine use of BHT in food chemistry is not encouraged anymore.

Because of its anti-oxidative properties [17,18], BHT can be used to interfere with free-radical-mediated reactions taking place in the lipid compartment of complex lipid–protein assemblies, such as biomembranes and lipoproteins. Since ferroptosis involves the iron-dependent lipid peroxidation of complex membrane lipids [1,15], BHT might interfere with ferroptotic signalling and thus might protect cells from ferroptotic cell death. In this study, we explored the impact of BHT on ferroptosis in cultured human neuroblastoma cells and found that SH-SY5Y cells are strongly protected from RSL3- and ML162-induced ferroptosis at sub-micromolar concentrations in a dose-dependent manner. RSL3-induced oxidation of the cellular membrane lipids was prevented by BHT, and in an in vivo Alzheimer’s disease model we observed that the upregulated expression of ferroptosis-related genes was normalised when the animals were pre-treated with BHT. From these data, it can be concluded that BHT might interfere with ferroptotic signalling and that Alzheimer’s patients might benefit from treatment with radical scavenging drugs.

## 2. Materials and Methods

### 2.1. Chemicals and Devices

The chemicals used were purchased from the following vendors: SH-SY5Y cells from the German Collection of Microorganisms and Cell Culture GmbH (DSMZ, Braunschweig, Germany); phosphate-buffered saline (PBS) from PAN Biotech (Aidenbach, Germany); cell culture materials and media from PAN Biotech (Aidenbach, Germany), authentic HPLC standards (15S/R-HETE, 12S/R-HETE, 5S/R-HETE) from Cayman Chem (distributed by Biomol GmbH, Hamburg, Germany); acetic acid from Carl Roth GmbH (Karlsruhe, Germany); sodium borohydride from Life Technologies, Inc. (Eggenstein, Germany); butylhydroxy toluene (BHT) from European Pharmacopoeia (Strasbourg, France). RSL3 and Ferrostatin-1 from Cayman (Ann Arbor, MI, USA), ML162 and Liproxstatin-1 from Tocris Bioscience (Bristol, UK). Oligonucleotide synthesis was performed at BioTez Berlin Buch GmbH (Berlin, Germany). HPLC-grade methanol, acetonitrile, n-hexane, 2-propanol, and water were from Fisher Scientific (Newington, NH, USA).

For HPLC analysis of the cellular lipids, a Shimadzu instrument (Shimadzu Germany, Berlin, Germany) involving two LC-20 AD pumps, a DGU-20A3 degassing unit, an SPD-M20A diode array detector, a CTO-20 AC column oven, a CBM-20A steering unit, and a SIL-20AC auto-injector were used.

### 2.2. Cell-Viability Assay (CCK8)

The cell-viability assessment was carried out using the CCK8 kit from Elabscience (Houston, TX, USA). SH-SY5Y cells are adherent cells, which rapidly grow to confluency. For our routine ferroptosis experiments, we suspended the cells in culture medium at a density of 3 × 10^5^ cells per mL (seeding density) and transferred 100 µL into the wells of a 96-well plate. The cells were then cultured to reach near confluency and then the experiment was started. In experiments, for which more cells are needed (HPLC analyses, GPX4 activity assays), we prepared a cell suspension with a seeding density of 10^6^ cells per mL and added 10 mL of this suspension to 10 cm Petri dishes or T75 cell culture flasks (10^7^ cells per dish). Afterwards, the cells were allowed to grow the near confluency and then the experiment was started. The pre-confluent cells were then pre-incubated for 2 h with different concentrations of BHT (0.03 µM to 30 µM) or other ferroptosis inhibitors of ferrostatin-1, liproxstatin-1, and then ferroptosis was induced by the addition of different concentrations of RSL3 or ML162. After a 24 h incubation period, cell viability was assayed as described in the CCK8 test kit manual using a microplate reader (Promega, Madison, WI, USA). The numbers of repetitions and statistical evaluations of the experimental raw data were performed as described in Section 2.8.

### 2.3. Lipid Extraction, Hydrolysis, and HPLC Analysis

For these experiments, SH-SY5Y cells were seeded into 10 cm Petri dishes at a density of 1 × 10^7^ cells per dish and then the cells were allowed to grow to near confluency. Then, BHT was added to reach a final concentration of 1 µM and the cells were incubated with BHT for two additional hours. Following this pre-incubation, ferroptosis was induced by the addition of 10 µM (final concentration) of RSL3 or ML162. After a 24 h incubation period, cells and cellular remnants were spun down (100.000 g), reconstituted in 1 mL of PBS, and the hydroperoxy lipids were reduced to the corresponding hydroxy compounds by the addition of solid sodium borohydride (1 mg/mL final concentration). Following sonication, 2.5 mL of methanol and 1.25 mL of chloroform were added, and the cellular lipids were extracted according to [26]. The solvents were evaporated, and the extracted lipids were reconstituted with 450 μL of anaerobic methanol. After the addition of 75 μL of anaerobic KOH (40%), the ester lipids were hydrolysed for 15 min at 60 °C. Then, the samples were cooled down on ice and 75 μL of concentrated acetic acid was added. Precipitate was removed by centrifugation and the clear hydrolysates were analysed by RP-HPLC to quantify the amounts of major PUFAs and their mono-hydroxylated derivatives. 

### 2.4. Measurements of the Cellular Oxidation Potential 

The cellular oxidation potential was quantified using the fluorescence detection kit from Kushan Zist (Tehran, Iran). This kit is based on the oxidation of the fluorescence indicator dichloro-dihydrofluorescein diacetate (DCF-DA) by intracellular oxidizing agents. The fluorescence indicator in its reduced form is taken up by cells but it is not fluorescence-active as long as it remains in its reduced form. However, when oxidized by intracellular oxidizing agents, such as reactive oxygen species, it becomes fluorescent and emits light when stimulated by irradiation (480–500 nm). For our experiments, SH-SY5Y cells were plated at a density of 3 × 10^4^ cells per well in the wells of a 96-well plate. After a 24 h culturing period, the cells reached near confluency and BHT (1 µM final concentration) was added. Then, the cells were pre-incubated with this compound for 2 h. Next, RSL3 (10 µM final concentration) was added to initiate ferroptosis. After 24 h, the cells were washed three times with PBS and incubated with 30 μM DCF-DA (redox-active fluorescence indicator) for 45 min at 37 °C in the dark. Finally, cells were washed again with PBS and resuspended in a buffer containing 130 mM NaCl, 5.4 mM KCl, 0.8 mM MgCl_2_, 1.8 mM CaCl_2_, 15 mM glucose, and 5 mM HEPES at pH 7.4. The relative levels of fluorescence were quantified by fluorescence spectrophotometer (microplate reader, Promega, Madison, WI, USA). The excitation wavelength was 480–500 nm, and the emission wavelength 510–550 nm.

### 2.5. RNA Extraction, Reverse Transcription and qRT-PCR of GPX4- and ALOX-Isoforms

SH-SY5Y cells were seeded in 6-well plates at a density of 1 × 10^6^ cells per well. After a 24 h culturing period, BHT was added at different concentrations and the cells were pre-incubated for 2 h. Subsequently, ferroptosis was induced by the addition of 10 µM (final concentration) of RSL3. After 24 h, total RNA was extracted using the Takara RNA extraction kit (Takara, Tokyo, Japan), and concentrations of the RNA preparations were determined. RNA was reversely transcribed using the First Strand cDNA Synthesis Kit (Takara, Kyoto, Japan) and for this purpose 0.1–2 µg of the total RNA preparation was employed. Quantitative real-time PCR (qRT-PCR) was carried out with a Rotor-Gene 3000 device (Corbett Research, Sydney, Australia) using the SensiFast SYBR PCR Kit (Bioline, Luckenwalde, Germany). The following primer combinations were employed for the different gene products. GAPDH: 5′-CCA TCA CCA TCT TCC AGG AGC GA-3′ (forward) and 5′-GGA TGA CCT TGC CCA CAG CCT TG-3′ (reverse); allGPX4: 5′-TGT GCG CGC TCC ATG CAC GAC T-3′ (forward) and 5′-CGA ATT TGA CGT TGT AGC CCG-3′ (reverse); mGPX4: 5′-CTC GGC CGC CTT TGC CGC CTA-3′ (forward) and 5′-CGA ATT TGA CGT TGT AGC CCG-3′ (reverse); nGPX4: 5′-CCG GCG GAA GAA GCC CTG TCC-3′ (forward) and 5′-CGA ATT TGA CGT TGT AGC CCG-3′ (reverse); ALOX15: 5′-ACT GAA ATC GGG CTG CAA GGG G-3′ (forward) and 5′-ACT GAA ATC GGG CTG CAA GGG G-3′ (reverse); ALOX15B: 5′-GTG CAG TGG AAC GCT TTG TCT C-3′ (forward) and 5′-AAG CAC AGG AGT CAA ACT GCC C-3′ (reverse); ALOX12: 5′-GGG CGA GGA GGA GGA GTT TGA T-3′ (forward) and 5′-TCC AGG TGG CCC AGC AGT AGA T-3′ (reverse); ALOX12B: 5′-TCC AGG TGG CCC AGC AGT AGA T-3′ (forward) and 5′-TCC AGG TGG CCC AGC AGT AGA T-3′ (reverse); ALOX5: 5′-GTG GCG CGG TGG ATT CAT A-3′ (forward) and 5′-GGG TTC CAC TCC ATC CAT CG-3′ (reverse); ALOXE3: 5′-GCC GGC ACA CTG GAC AAC ATC-3′ (forward) and 5′-CAC GGT GCA GTA GCC TTC AAT CC-3′ (reverse). The amplification protocol involved the following steps: denaturation phase (15 s at 95 °C), annealing phase (30 s at 65 °C), and synthesis phase (20 s at 72 °C). Forty denaturation–annealing–synthesis cycles were usually run and melting curves of the amplification products were recorded online. The Rotor-Gene Q software (6.07) was used to analyse the PCR raw data. Specific amplicons were prepared as external amplification standards for all mRNA targets and for the reference mRNA (GAPDH), and this procedure allowed for the exact quantification of the copy numbers of the target mRNA species in our RNA preparation. Finally, we calculated the copy numbers of target mRNA per 10^6^ GAPDG mRNA copies.

### 2.6. In Vitro GPX4 Activity Assay

To assay the catalytic activity of GPX4 in SH-SY5Y cells, about 10^7^ cells were seeded into T75 cell culture flasks (Sarstedt, Nümbrecht, Germany). After a 24 h culturing period, the cells reached near-confluency and BHT was added to reach a final concentration of 1 µM. Then, the cells were pre-incubated for 2 h. After the pre-incubation period, RSL3 was added at a final concentration of 10 µM and cells were kept in culture for an additional 24 h. After this, the cells were harvested and reconstituted in 200 µL of cell lysis buffer (50 mM Tris-HCL, pH 7.5 containing 1% Triton X100, 10% glycerol, 0.3 M KCl, protease inhibitor, 5 mM TCEP, 0.1 mM EDTA). The cellular lysates were centrifuged (20,000× g) and aliquots of the cellular lysate supernatants were added to the activity assay mixture.

The reaction buffer for the activity assays was a 100 mM Tris-HCl buffer (pH 7.5) involving 5 mM EDTA, 1% Triton-X100, 2 IU of glutathione reductase 3 mM glutathione, and 0.2 mM NADPH. For the measurements, 50 μL of the cell lysate supernatant was mixed with 445 µL of the reaction buffer and pre-incubated for 1 min. Then, the GPX4 reaction was started by the addition of a small aliquot (10 µL) of a methanolic stock solution of phospholipid hydroperoxides (50 µM final concentration in the assay mixture), which was prepared with soybean lipoxygenase-1 [27] and purified by RP-HPLC. As primary readout parameter, the decrease in absorbance at 340 nm was assayed using the Shimadzu uv/vis spectrophotometer UV-1900i. The GPX4 activity was calculated in nmoles of consumed NADPH per mg of lysate supernatant protein per second using a molar extinction coefficient of NADPH of 6.22 × 10^3^ (M × cm)^−1^.

### 2.7. In Vivo Rat Ferroptosis Model

To test whether BHT may also impact ferroptosis in vivo, we employed the rat streptozotocin-induced Alzheimer’s disease model. This model has frequently been used in the literature [28,29,30]. It involves the stereotactic intraventricular application of streptozotocin and induces functional AD symptoms such as cognitive impairment and memory defects. Streptozotocin disrupts insulin-signalling in the brain and induces oxidative stress, inflammation, and neurodegeneration, which are canonic clinical symptoms of human AD. In other words, the rat streptozotocin AD model mirrors important functional characteristics of human AD, but the morphological AD characteristics such as extracellular Aβ plaque formation and the intracellular deposition of hyperphosphorylated tau proteins (NFT) are less adequately mirrored.

The in vivo rat experiments (Alzheimer’s disease model, preparation of hippocampus, RNA extraction, semi-quantitative RT-PCR) were carried out at the University of Tehran in strict compliance with the Animal Ethics guidelines of this university (allowance certificate IR.UT.SCIENCE.REC.1401.015, University of Tehran). For the experiments, healthy male albino Wistar rats with an initial body weight ranging between 180 and 200 g were used. The rats were housed at the Central Animal House of the Medical Physiology Department at the University of Tehran. Throughout the treatment and the surgical procedures, the rats were maintained under controlled environmental conditions, including a room temperature of 25 ± 2 °C, a relative humidity between 45 and 55%, and a 12 h light/dark cycle. They had unrestricted access to a standard rodent-pelleted diet and water. Moreover, the rats were fasted for 12 h before surgical intervention.

To explore the impact of BHT (Sigma, Deisenhofen, Germany) on the expression of AD-related genes, a total of 30 rats were grouped into 3 experimental categories each consisting of 10 individuals. Sham group: These animals received BHT at a dose of 120 mg/kg body weight. They underwent surgery but PBS was injected intraventricularly instead of the streptozotocin solution. AD group: These animals did not receive any BHT. They underwent surgery and a streptozotocin solution was injected intraventricularly. AD + BHT group: These animals received BHT at a dose of 120 mg/kg body weight. They underwent surgery and streptozotocin was injected intraventricularly to induce AD symptoms.

To induce the development of AD-related symptoms, animals were anesthetised intraperitoneally with a mixture of ketamine (100 mg/kg, i.p.) and xylazine (10 mg/kg, i.p.). They were then placed on a stereotaxic frame. Intraventricular injections of a streptozotocin solution were carried out with a 5 μL Hamilton micro-syringe. AD and AD + BHT groups received bilaterally 3.0 mg streptozotocin (Sigma-Aldrich, Saint Louis, United States) per kg body weight dissolved in PBS (2.5 μL each site). After surgery, the rats were kept in separate cages for recovery in a well-ventilated room at 25 °C. In this in vivo rat model of AD, the animals developed AD-related symptoms approximately 3 weeks after surgery [31].

For RT-PCR quantification of AD-related gene products, three animals from each group were sacrificed, the brain was perfused with PBS, and the hippocampus was prepared. Total RNA was extracted with the RNA extraction kit RiboEx^TM^ (GeneAll Biotechnology, Seoul, Republic of Korea). RNA extracts were quantified and aliquots were reversely transcribed using reverse transcriptase (Parstus Biotechnology, Tehran, Iran) and semi-quantitative RT-PCR (Qiagen Rotor Gene, Hilden, Germany) was carried out for three different AD-related genes (*Alox15*, *Acsl4*, *Fth1*) and the reference gene (Gapdh) using the Power SYBR™ Green PCR Master Mix (Amplicon, Copenhagen, Denmark). For this purpose, PCR samples were first incubated for 2 min at 50 °C and then for 2 min at 95 °C. After this pre-treatment, 40 amplification cycles were run, each consisting of a denaturation phase (15 s at 95 °C), an annealing phase (60 s at 60 °C), and a synthesis phase (1 min at 72 °C). The relative expression of the target genes (*Alox15*, *Acsl4*, *Fth1*) was normalised for *Gapdh* gene expression and the ΔΔC_t_ method was used for quantification of the relative expression levels. The following primer pairs were employed for the amplification protocol. *Acsl4*: AAA ATG AAG TTG AGC CTG TCG (forward), GCC ACC GAT CAC AAT CTCA (reverse); *Alox15*: TGG CTG CAC CGT GGT TG (forward), CAG TTG CCC CAC CTG TAC AGA (reverse); *Fth1*: GCC AGA ACT ACC ACC AGG AC (forward), CGG TCA AAA TAA CAA GAC ATG G (reverse); and *Gapdh*: GGT GAA GGT CGG TGT GAA C (forward), TTG TCA CAA GAG AAG GCA GC (reverse).

### 2.8. Numbers of Repetitions and Statistic Evaluation of the Experimental Raw Data 

Statistical calculations and figure design were performed using GraphPad Prism version 8.00 for Windows (GraphPad Software, La Jolla, CA, USA). The experimental data shown in each figure originate from single experiments, which were designed on the outcome of a number of preliminary experiments testing the optimal experimental conditions, such as cell number, incubation time, pre-incubation period, concentrations of additives, and others. 

**Figure 1:**
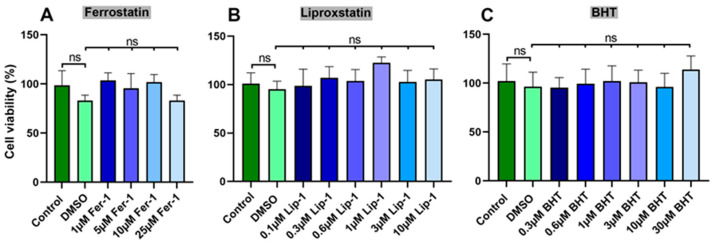
Standard ferroptosis inhibitors and BHT do not impact the in vitro cell viability of SH-SY5Y neuroblastoma cells. SH-SY5Y neuroblastoma cells were seeded in 96-well plates at a density of 30,000 cells/well (0.1 mL of culture medium) and were cultured under standard conditions to near-confluency in the presence and absence of different concentrations of ferrostatin-1 (**A**), liproxstatin-1 (**B**) and BHT (**C**). Cell viability was assayed as described in Section 2.2. Four independent wells (*n* = 4, four biological replicates) of a 96-well plate were set-up for each experimental group. Means ± SD were determined. For significance calculations, normal distribution of the numeric values was tested and one-way ANOVA was performed to obtain *p*-values. ns, not significant.

**Figure 2:**
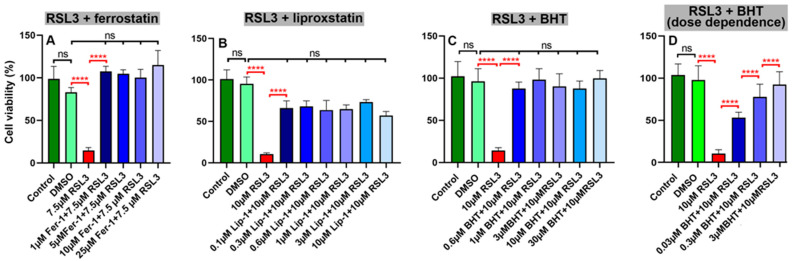
The standard ferroptosis inhibitors ferrostatin-1 and liproxstatin-1 but also BHT prevented RSL3-induced ferroptosis in SH-SY5Y neuroblastoma cells. SH-SY5Y cells were cultured in 96-well plates to near-confluency. Then, the cells were pre-incubated for 2 h with different concentrations of the standard ferroptosis inhibitors ferrostatin-1 and liproxstatin-1 as well as with BHT. Finally, ferroptosis was induced by the addition of RSL3, and after a 24 h incubation period cell viability was assayed as described in Section 2.2. (**A**) RSL3 + ferrostatin-1; (**B**), TSL3 + liproxstatin-1; (**C**) RSL3 + BHT; (**D**) RSL3 + BHT, dose-dependence. Four independent wells (*n* = 4, four biological replicates) of a 96-well plate were set up for each experimental group. Means ± SD were determined. For significance calculations, normal distribution of the numeric values was tested and one-way ANOVA was performed to obtain *p*-values. ****, *p* < 0.001; ns, not significant.

**Figure 3:**
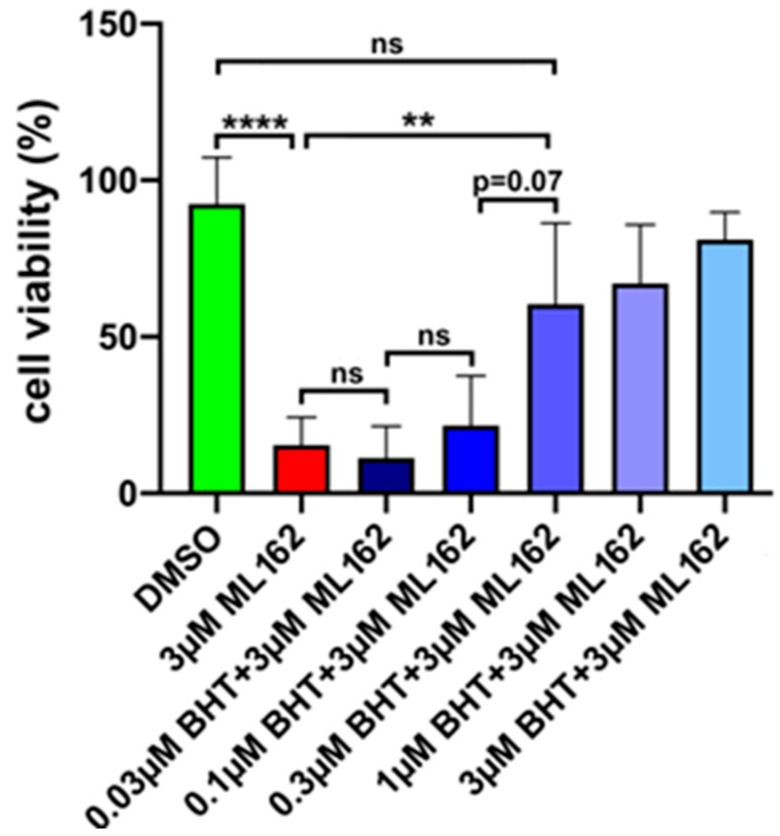
BHT prevented ML162-induced ferroptosis in SH-SY5Y neuroblastoma cells. SH-SY5Y cells were cultured in the wells of a 96-well plate to near-confluency. Then, the cells were pre-incubated for 2 h with different concentrations of BHT. Finally, ferroptosis was induced by the addition of ML162, and after a 24 h incubation period cell viability was assayed as described in Section 2.2. Four independent wells (*n* = 4, four biological replicates) of a 96-well plate were set-up for each experimental group. Means ± SD were determined. For significance calculations, normal distribution of the numeric values was tested and one-way ANOVA was performed to obtain *p*-values. ****, *p* < 0.0001; ** *p* < 0.01; ns, not significant.

For the cell survival experiments (Figure 1, Figure 2 and Figure 3), four independent wells of a 96-well plate were set up for each experimental group representing each column in the images. Means ± SD were determined. For significance calculations, normal distribution of the numeric values was tested and one-way ANOVA was performed to obtain *p*-values.

**Figure 4:**
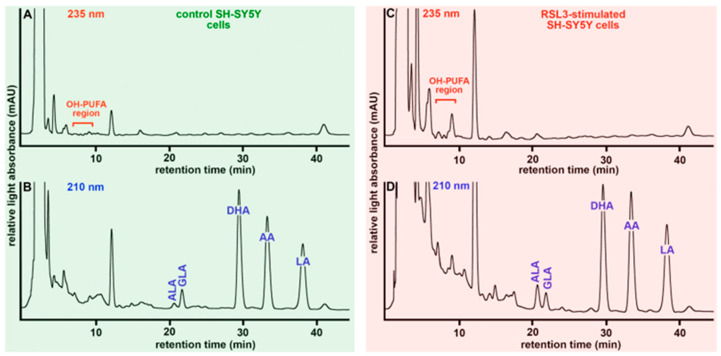
RP-HPLC analysis of the oxidation degree of SH-SY5Y membrane lipids. SH-SY5Y cells were grown to near-confluency in 10 cm Petri dishes and ferroptosis was induced by the addition of 10 µM RSL3. After a two-hour incubation period, the cells were harvested, washed, and the membrane lipids were extracted according to Bligh–Dyer [26]. The solvents were evaporated, the remaining membrane lipids were hydrolysed under alkaline conditions, and the free fatty acid derivatives were analysed by RP-HPLC (see Section 2.3.) following the chromatograms simultaneously at 235 nm (oxygenated PUFA derivatives) and 210 nm (non-oxidised PUFAs). (**A**) Analysis of the hydroxy-PUFA of confluent SH-SY5Y cells cultured in the absence of RSL3. (**B**) Analysis of non-oxidized PUFAs of SH-SY5Y cells cultured in the absence of RSL3. (**C**) Analysis of hydroxy-PUFA of confluent SH-SY5Y cells cultured for 2 h in the presence of RSL3. (**D**) Analysis of non-oxidised PUFAs of SH-SY5Y cells cultured for 2 h in the presence of RSL3. ALA, alpha-linolenic acid; GLA, gamma-linolenic acid; AA, arachidonic acid; LA, linoleic acid; DHA, docosahexaenoic acid. Four independent cell cultures (*n* = 4, four biological replicates) were set up for each incubation condition (absence of RSL3 vs. presence of RSL3) and representative chromatograms are shown. Example chromatograms for the other experimental groups (SH-SY5Y cells + BHT, SH-SY5Y cells + 1 µM BHT + 10 µM RSL3) are shown in Supplementary Figure S1.

**Figure 5:**
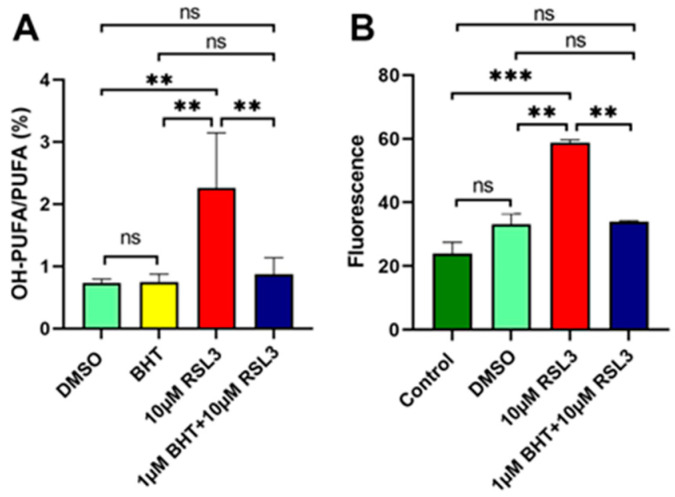
BHT prevented RSL3-induced oxidation of membrane lipids. (**A**) RP-HPLC quantification of the OH-PUFA/PUFA ratio as suitable readout parameter characterising the oxidation degree of the cellular membrane lipids. Panel (**A**): SH-SY5Y cells were cultured in 10 cm Petri dishes in the absence (solvent control, DMSO, green column) and presence of 10 µM RSL3 (red column). After reaching near-confluency, 1 µM BHT was added and the cells were further cultured for 2 h. Then, cells were harvested, membrane lipids were extracted, the ester lipids were hydrolysed, and the hydrolysates were analysed by RP-HPLC as described in Section 2.3. Four different Petri-dishes (*n* = 4, four biological replicates) were set up for each of the four different experimental groups (control cells, BHT-treated cells, RSL3-treated cells, and BHT- + RSL4 treated cells). All samples were worked up and analysed separately. Means ± SD were determined and significance calculations were carried out as described above for the cellular survival experiments. ** *p* < 0.01; ns, not significant. Panel (**B**): For measurements of the cellular oxidation potential, SH-SY5Y cells were seeded into the wells of a 96-well plate, and for each experimental group three different wells (*n* = 3, three biological replicates) were used. After the work-up procedure the fluorescence of the cells of each well was quantified separately. Means ± SD were determined and significance calculations were carried out as described above for the cellular survival experiments. ** *p* < 0.01; *** *p* < 0.001; ns, not significant.

For the HPLC analyses (Figure 4 and Figure 5A), four different Petri dishes were set up for each of the four different experimental groups (control cells, BHT-treated cells, RSL3-treated cells, BHT- + RSL4 treated cells). All samples were worked up and analysed separately and no sample pooling was carried out. Here again, means ± SD were determined and significance calculations were carried out as described above for the cellular survival experiments. For measurements, the cellular oxidation potential (Figure 5B) cells were seeded into the wells of a 96-well plate, and for each experimental group (column) three different wells were used. After the work-up procedure the fluorescence of the cells of each well was quantified separately.

**Figure 6:**
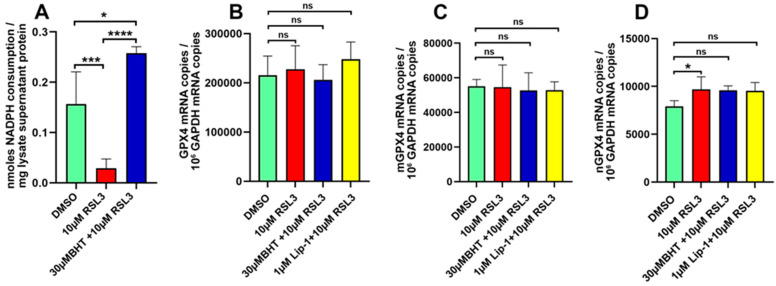
Expression of GPX4 isoforms in control and ferroptotic SH-SY5Y cells. Panel (**A**): GPX activity assays. SH-SY5Y cells were cultured to near-confluency in T75 cell-culture flasks, and for each experimental group two separate culture flasks (two biological replicates) were set up. Then, cells were pre-incubated in the presence and absence of BHT and after a 2 h pre-incubation period ferroptosis was induced by the addition of RSL3. Cells from each flask were disrupted separately, the lysates were centrifuged, and two independent GPX4 activity measurements (two technical replicates) were carried out with each lysate using hydroperoxy phospholipids as substrate. Means ± SD were calculated and one-way ANOVA was carried out. Panels (**B**–**D**): Expression of GPX4-isoforms. SH-SY5Y cells were cultured as described in the legend to Panel A (two biological replicates). After reaching near-confluency, cells were pre-incubated with ferroptosis inhibitors Lip-1 or BHT. After a 2 h pre-incubation period, ferroptosis was induced by the addition of RSL3. Cells were prepared, total RNA was extracted, and the steady state concentrations of the different GPX4 isoforms were determined by qRT-PCR using isoform-specific primer combinations (see Section 4 for methodological details and primer sequences). Two separate qRT-PCR runs (two technical replicates) were run for each mRNA extract. (**B**) Quantitative RT-PCR of the mRNA species encoding for all GPX4 isoforms (cytosolic GPX4, mitochondrial GPX4, nuclear GPX4). (**C**) Quantitative RT-PCR of the mRNA species encoding for mitochondrial GPX4 only. (**D**) Quantitative RT-PCR of the mRNA species encoding for the nuclear GPX4 only. *, *p* < 0.05; ***, *p* < 0.001; **** *p* < 0.0001; ns, not significant.

For the subcellular GPX4 activity assays (Figure 6A), SH-SY5Y cells were cultured in T75 cell-culture flasks, and for each experimental group (each column in the image) two separate culture flasks were used. Cells from each flask were disrupted separately (not pooled); the lysates were centrifuged and two independent GPX4 activity measurements were carried out with each lysate. Means ± SD were calculated and one-way ANOVA was carried out to obtain the numeric *p*-values.

For the gene expression studied (Figure 6B–D), SH-SY5Y cells were cultured to near-confluency in the wells of six-well plates, and two wells were set up for each experimental group. RNA was separately extracted from the cells of each well, and for each RNA extract two different qRT-PCR samples were run. Expression levels of the target genes were normalized for GAPDH expression and means ± SD were determined. Significance calculations were performed with one-way ANOVA.

**Figure 7:**
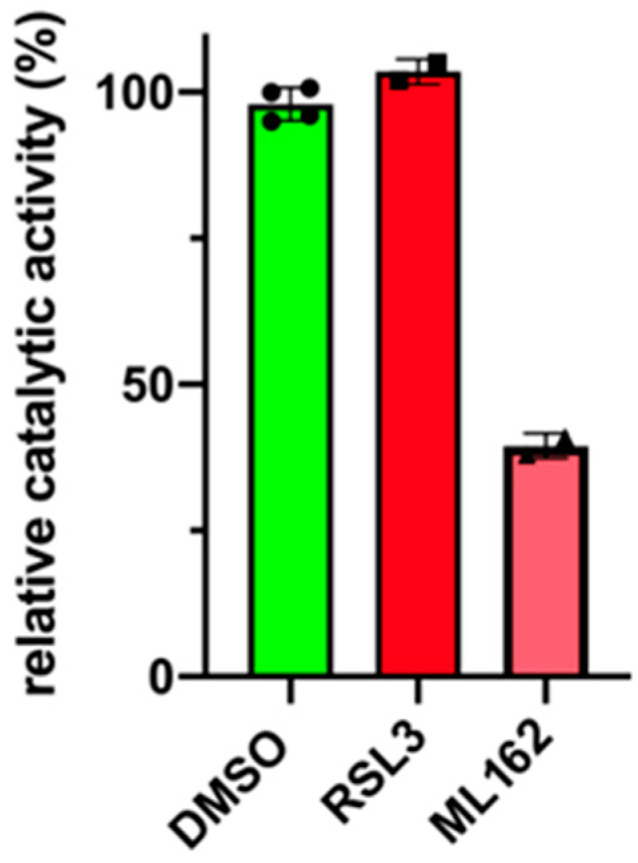
RSL3 does not inhibit the catalytic activity of recombinant selenium-containing human GPX4. Expression of recombinant selenium-containing human GPX4 and in vitro GPX4 activity assays were carried out as described in Section 2.6. Aliquots of the recombinant enzyme preparation were pre-incubated in the assay mixture with 20 µM RSL3 and ML162 for 1 h, and then the GPX4 reaction was started by the addition of *tert*-butyl hydroperoxide as substrate. The decrease in absorbance at 340 nm was assayed. Four independent activity measurements (*n* = 4, four technical replicates) were carried out for the DMSO control and two activity assays (*n* = 2, two technical replicates) were performed for each ferroptosis inducer (RSL3, ML162). Each symbol represents an individual measurement. A single enzyme preparation of recombinant selenocysteine-containing human GPX4 was employed. The mean of the DMSO activity assays was set at 100%.

For the in vitro activity assays of recombinant human GPX4 (Figure 7), four independent activity measurements were carried out for the DMSO control and two activity assays were performed for each ferroptosis inducer (RSL3, ML162). A single enzyme preparation of recombinant selenocysteine-containing human GPX4 was employed.

**Figure 8:**
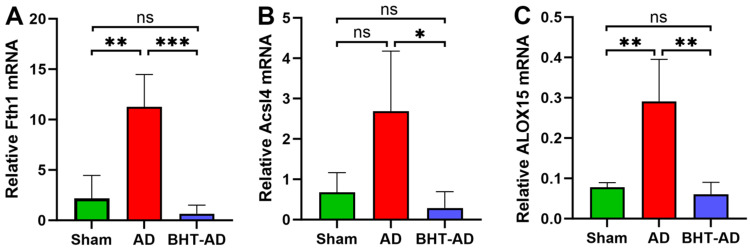
BHT prevents the upregulation of ferroptosis-related genes in an in vivo model of Alzheimer’s disease. Rats were orally pre-treated with BHT at a dose of 120 mg/kg body weight for 4 days and then the formation of AD-related symptoms was induced by bilateral intracerebroventricular injections of streptozotocin (3 mg/kg on both sides). Hippocampal tissue was prepared, RNA was extracted, and semi-quantitative RT-PCR of selective Alzheimer’s disease-related genes was carried out. Three different rats (*n* = 3, three biological replicates) were set-up for each of the three experimental groups. After appropriate treatment, the hippocampus was prepared from each rat, RNA was extracted, and two independent RT-PCR runs were carried out with each RNA extract. Means ± SD were quantified and one-way ANOVA was used to calculate the *p*-values. (**A**) *Fth1* expression, (**B**) *Acsl4* expression, (**C**) *Alox15* expression. * *p* < 0.05, ** *p* < 0.01, *** *p* < 0.001, ns, not significant.

For the in vivo experiments (Figure 8), three different rats were set up for each of the three experimental groups (three different columns in the image). After appropriate treatment, the hippocampus was prepared from each rat, RNA was extracted, and two independent RT-PCR runs were carried out with each RNA extract. Means ± SD were quantified and one-way ANOVA was used to calculate the *p*-values. The degree of statistical significance was always defined the same way: * *p* < 0.05, ** *p* < 0.01, *** *p* < 0.001, **** *p*< 0.0001.

## 3. Results

### 3.1. Butylated Hydroxytoluene Does Not Impact the Viability of Human Neuroblastoma Cells 

Butylated hydroxytoluene (BHT) is a powerful radical scavenger that prevents free radical-mediated lipid peroxidation [17]. Since ferroptosis involves lipid peroxidation as a crucial step, we reasoned that BHT might prevent ferroptotic cell death. To test this hypothesis, we first explored whether standard ferroptosis inhibitors such as ferrostatin-1 and liproxstatin-1 [32] but also BHT might impact the viability of human neuroblastoma cells (SH-SY5Y).

For this purpose, SH-SY5Y cells were seeded in the wells of 96-well plates at a density of 30,000 cells/well (cells were resuspended in 0.1 mL of cell culture medium) and cultured under standard conditions in the presence and absence of different concentrations of ferrostatin-1, liproxstatin-1, and BHT to near-confluency. From Figure 1, it can be seen that neither of these compounds significantly altered cell viability. Even at concentrations in the two-digit micromolar range, cell viability was similar to that of the solvent control (DMSO) experiments.

### 3.2. Butylated Hydroxytoluene Protects Human Neuroblastoma Cells from RSL-3-Induced Ferroptosis

To explore whether ferroptosis can be induced in SH-SY5Y cells, we incubated the cells in the presence and absence of the ferroptosis-inducer RSL3. This compound interferes with cellular glutathione peroxidase 4 (GPX4) and thus induces ferroptotic cell death [33,34,35]. When we cultured confluent SH-SY5Y cells in the presence of 7.5 µM of RSL3 (Figure 2A), we observed a strong reduction in cell viability. This effect was prevented when the cells were pre-incubated with the standard ferroptosis-inhibitors ferrostatin-1 (Figure 2A) and liproxstatin-1 (Figure 2B). Most interestingly, BHT also prevented RSL3-induced ferroptosis (Figure 2C) at concentrations as low as 0.6 µM.

To test whether the protective effect of BHT was dose-dependent, we carried out an additional experiment using 10 µM of RSL3 as a ferroptosis inducer and employed different BHT concentrations to rescue the cells. Here, we observed that the pre-incubation of the cells with BHT prevented RSL3-induced ferroptosis in a dose-dependent manner with an approximate IC_50_ of about 30 nM (Figure 2D).

### 3.3. Butylated Hydroxytoluene Also Protects SH-SY5Y Cells from ML162-Induced Ferroptosis

RSL3 is the most frequently used ferroptosis-inducer in cellular systems, but alternative compounds are also available. ML162 is one of these compounds [36], and we explored whether ML162-induced ferroptosis can also be inhibited by BHT.

As indicated in Figure 3, ML162 induces ferroptosis in SH-SY5Y cells at one-digit micromolar concentrations. When cells were pre-incubated with different concentrations (0.03–3 µM) of BHT, the degree of ferroptosis was reduced in a dose-dependent manner, and at 3 µM BHT complete prevention was observed. These data indicate that BHT prevented ferroptosis independent of the chemical properties of the ferroptosis inducer.

### 3.4. Butylated Hydroxytoluene Prevented RSL3-Induced Oxidation of Membrane Lipids

When membrane lipids are oxidised by non-enzymatic and/or enzymatic processes, the membrane structure is disturbed, which might impair the barrier function of the membrane. This has previously been reported for endoplasmic membranes [37] but also for the plasma membrane of human erythrocytes [38]. To test whether RSL3-treatment impacts the oxidation of the membrane lipids, we incubated SH-SY5Y cells for 24 h in the presence and absence of RSL3, prepared the cellular membranes, extracted the membrane lipids, hydrolysed them under mild alkaline conditions, and analysed the hydrolysates by RP-HPLC to quantify both the cellular content of polyunsaturated fatty acids (PUFA) and of the oxygenated PUFAs (OH-PUFAs). From these two primary readout-parameters, we calculated the OH-PUFA/PUFA ratio (in %), which quantifies the degree of oxidation of the membrane lipids [39,40]. Example chromatograms of the membrane lipids prepared from solvent control cells (Figure 4A,B) and from RSL3 treated cells (Figure 4C,D) are shown. For better comparison, we normalised the intensity scale of the chromatograms for the most abundant PUFA (linoleic acid) peak. Following the chromatograms at 210 nm (Figure 4B,D), we quantified the non-oxidised PUFAs, and these data show that arachidonic acid (AA) and linoleic acid (LA) are the dominant PUFAs in the cellular membrane lipids. Smaller amounts of alpha-linolenic acid (ALA) and gamma-linolenic acid (GLA) were also detected. Interestingly, the relative contributions of DHA, AA, LA, and GLA to the sum of the major PUFAs were similar in control to SH-SY5Y cells (Figure 4B) and RSL3-treated cells (Figure 4D). In contrast, the relative share of ALA was lower in control cells when compared with the RSL3-treated cells. The mechanistic basis for this observation has not been explored in detail, but it might be possible that RSL3-treatment stimulates intracellular ALA synthesis. Alternatively, ALA might be protected from RSL3-induced lipid peroxidation so that its relative share is elevated after RSL3-treatment.

Comparing the chromatograms recorded at 235 nm of RSL3-treated (Figure 3C) and control SH-SY5Y cells (Figure 3A), we found higher levels of oxygenated PUFAs (higher peaks in the OH-PUFA region) in the RSL3 cells. 

Finally, we calculated OH-PUFA/PUFA ratios (in %) from the RP-HPLC chromatograms as suitable measures for the oxidation degree of the membrane lipids [34,35], and these data are summarized in Figure 5A. Under basic conditions (no RSL3 treatment), the OH-PUFA/PUFA ratio of the membrane lipids was 0.73 ± 0.06% (Figure 5A, green column), and these data (four independent cell preparations) indicate that about seven OH-PUFA residues were present in the membrane lipids per 1000 PUFA residues. These data indicate that the degree of membrane lipid oxidation was low in resting SH-SY5Y cells. When the cells were cultured in the presence of BHT (1 µM), the OH-PUFA/PUFA ratio was hardly altered (Figure 5A, yellow column). However, when the cells were incubated in the presence of RSL3 (Figure 5A, red column), the OH-PUFA/PUFA ratios went up to 2.26 ± 0.89%, indicating a three-fold increase in the degree of membrane lipid oxidation. In the presence of BHT (Figure 5A, blue column), this increase was completely prevented (Figure 5A, blue column). Taken together, these data indicate that BHT prevented RSL3-induced membrane lipid oxidation.

The elevated OH-PUFA/PUFA ratio measured in RSL3-traeted SH-SY5Y cells suggests an increased cellular oxidation potential, which might be related to an increased formation of reactive oxygen species (ROS). The cellular oxidation potential can easily be quantified using membrane-permeable redox-sensitive fluorescence probes [41]. For our study, we used the fluorescence-indicator dichloro-dihydrofluorescein diacetate (DCF-DA), which is membrane permeable and becomes fluorescent when converted intracellularly to its oxidized form. DCF-DA oxidation can be induced by reactive oxygen species (ROS), but also by other oxidizing agents that are formed during ferroptotic signalling. When we used this method (Figure 5B), we found that RSL3-treatment increased the intracellular fluorescence levels by a factor of two (Figure 5B, red column), and BHT (Figure 5B, blue column) prevented the RSL3-induced increase. In other words, the alterations in the intracellular oxidation potential parallels the oxidation of the membrane lipids.

### 3.5. RSL3 Inhibits Intracellular GPX4 Activity but Does Not Modify GPX4 Gene Expression

Glutathione peroxidase 4 (GPX4) [42] is a key player in ferroptotic signalling [1,2,3]. It reduces organic and inorganic peroxides to the corresponding alcohols and thus prevents free radical-mediated secondary reactions [42]. RSL3 induces ferroptosis by inhibiting the catalytic activity of the enzyme in cellular systems and thus drives cells into ferroptotic cell death [33,34]. On the other hand, in vitro GPX4 activity assays carried out with selenium-containing recombinant GPX4 did not inhibit the catalytic activity of human GPX4 [43]. When we assayed the activity of GPX4 using a cellular lysate of SH-SY5Y cells as enzyme source using hydroperoxy phospholipids as substrate, we found that RSL3-treatment strongly reduced the catalytic activity of this enzyme (Figure 6A, green vs. red columns). Interestingly, the RSL3-induced decline in catalytic activity was prevented when the cells were pre-treated with BHT (Figure 6A, red vs. blue column). The molecular basis for the protective effect of BHT has not been explored, but possible mechanisms are discussed in Section 4.

Next, we investigated whether or not the cellular mRNA concentrations encoding for the different GPX4 isoenzymes were modified by RSL3. Here, we found that the steady-state mRNA concentration of any GPX4 isoform was neither modified by RSL3, liproxstatin, nor BHT (Figure 6B–D), and these data indicate that GPX4 expression was hardly modified by these compounds.

### 3.6. RSL3 Does Not Inhibit Recombinant Selenium Containing GPX4 

RSL3 is a frequently employed inducer of ferroptosis [33,34,44], and mechanistic experiments suggested that this compound inhibits the catalytic activity of this enzyme [45]. To test the putative inhibitory effect of RSL3 on recombinant selenium containing GPX4, we pre-incubated the purified enzyme with RSL3 for 15 min and found that the compound did not inhibit the catalytic activity of the recombinant enzyme (Figure 7). This result is consistent with a recent literature report [45]. In addition, we also tested the GPX4 inhibitory activity of ML162 [46]. Here, we observed a 60% inhibition of the recombinant selenocysteine-containing enzyme. 

### 3.7. Lipoxygenase Isoforms Are Neither Expressed in Resting nor in RSL3-Treated SH-SY5Y Cells

Arachidonic acid lipoxygenases (ALOX-isoforms) are lipid-peroxidizing enzymes, which have been implicated in the mechanisms of ferroptosis [47,48,49]. To explore the expression patterns of the six human ALOX isoforms under basal conditions in SH-SY5Y cells, we employed a quantitative reverse transcriptase polymerase chain reaction (qRT-PCR) that involved the use of external amplification standards for each ALOX-isoform and for the reference enzyme GAPDH. This method allowed for the precise quantification of the cellular steady-state mRNA concentrations of each target enzyme, which is given in the number of mRNA copies of the ALOX-isoforms per 10^6^ GAPDH mRNA copies. Unfortunately, we did not obtain specific amplification signals. When we repeated the measurements with RNA extracts of RSL3-treated cells, we obtained similar results. Taken together, these data indicate that the steady-state ALOX mRNA isoforms were below the detection limits of our qRT-PCR system, which were about 10 copies of ALOX mRNA per 10^6^ GAPDH mRNA copies.

### 3.8. Oral Application of BHT Normalizes the Expression of Ferroptosis-Related Genes in a Rat Alzheimer’s Disease Model

Alzheimer’s diseases (AD) is the most prevalent neurodegenerative disease worldwide and has a big socio-economic impact [50,51]. In its early stages, it is characterised by neuronal dysfunction, which finally leads to neuronal cell death, and ferroptosis is one of the major cell-death mechanisms in the brains of AD patients [52,53]. To explore whether BHT is capable of preventing ferroptotic cell death in vivo, we orally pre-treated rats with BHT at a dose of 120 mg/kg body weight for 4 days. The low acute toxicity of BHT [20] allowed for the application of this dose, and previous results indicated that rats do not develop any symptoms of toxicity when treated this way [20]. Although we did not quantify any blood parameter to search for malfunctions of specific organs, we did not observe major signs of acute toxicity when we evaluated the overall health status (daily inspections) of the animals. To induce the formation of AD-related symptoms, streptozotocin at a dose of 3 mg/kg was locally administered through bilateral intracerebroventricular injections on both sides [54]. After surgery, the rats were allowed to recover for three weeks, and then the animals were sacrificed. The hippocampal regions were prepared and the expression of three AD-related gene products (Fth1, Acsl4, Alox15) were quantified by semi-quantitative RT-PCR.

Ferritin (Fth1) is an intracellular iron-binding protein [55], the expression of which is upregulated when cells are about to undergo ferroptosis [56]. We found a significant upregulation of Fth1 expression in streptozotocin-treated rats when compared with Sham-operated animals. Interestingly, this upregulation was prevented when the animals received BHT four days before streptozotocin installation. Fatty acid CoA ligase 4 (FACL4) is an enzyme that is encoded by the ACSL4 gene. It catalyses the formation of arachidonyl-CoA and thus facilitates the incorporation of this PUFA into the cellular ester lipid fraction. It has been implicated in ferroptotic signalling [57] and is frequently used in cellular systems as a ferroptosis marker. We observed that intraventricular streptozotocin installation upregulated the expression of Acsl4 mRNA (Figure 8B). Although this upregulation did not reach the level of statistical significance, it was completely prevented by BHT. Arachidonic acid 15 lipoxygenase (Alox15) has also been implicated in ferroptotic signalling [49]. Unfortunately, in the rat hippocampus, this lipid peroxidizing enzyme is only expressed at rather low levels (Figure 8C). However, streptozotocin installation significantly elevated the hippocampal Alox15 mRNA concentrations, and BHT treatment completely prevented this elevation.

In additional experiments, we also quantified the expression of the amyloid precursor protein (APP), which is of relevance for the pathogenesis of AD. In brain cells, the APP protein is metabolized by secretases forming the Aß-cleavage peptides. In AD patients, these cleavage peptides aggregate extracellularly to form the characteristic Aß plaques [58]. When we quantified the tissue concentrations of the APP mRNA in the hippocampus (Supplementary Figure S2), we obtained specific amplification signals. These signals were more intense after the induction of the AD symptoms although the difference between the two groups did not reach the level of statistical significance. Most interestingly, however, BHT treatment prevented the increase in APP expression and even reduced the basic expression of this gene (Sham group). Statistic evaluation revealed a significant difference (*p* < 0.05) between the AD rats (no BHT administration) and the HBT-treated AD animals (Supplementary Figure S2).

Taken together, our expression data suggest that ferroptosis-relevant genes are upregulated in this in vivo Alzheimer’s disease model and that this upregulation is prevented by BHT. Thus, BHT does not only prevent ferroptosis in our cellular in vitro systems but also in this in vivo model of Alzheimer’s disease.

## 4. Discussion

### 4.1. BHT Does Not Impact the Viability of SH-SY5Y Cells under Baseline Conditions but Protects Cells from RSL3-Induced Ferroptosis

2,6-Di-tert-butyl-4-methylphenol (BHT) is a powerful anti-oxidant which is frequently used as a preservative in lubricants and embalming fluids but also in cosmetics [17]. On the other hand, previous reports suggested that BHT may exhibit deleterious effects. A few reports suggested mutagenic and genotoxic activities of BHT [23,24,25], but more detailed investigations into these effects suggested that BHT does not represent a relevant mutagenic/genotoxic risk to man [23]. Some of the deleterious activities have been related not to BHT itself but to BHT metabolites. The pharmacological properties of some of these BHT metabolites have been explored [59], and most of them do not exhibit toxic effects. However, because of the complexity of the metabolite pattern, it still might be possible that some of the minor metabolites might exhibit toxic properties, and thus the use of BHT in food chemistry is problematic.

Because of its strong anti-oxidative properties, we tested the anti-ferroptotic properties of BHT in an in vitro cell culture model. For these experiments, we used the compound at concentrations of up to 30 µM but did not observe any signs of cell toxicity. Under the microscope, the cells looked normal and we did not observe any impact of BHT on cell viability. However, when we pre-incubated the cells with BHT and then induced ferroptosis by two different ferroptosis inducers (RSL3, ML162), we induced the resistance of the cells for this type of programmed cell death (Figure 2C,D and Figure 3A,B). The effects were dose-dependent and they appeared at sub-micromolar BHT concentrations. For RSL3-induced ferroptosis an IC_50_ in the two-digit nM range was estimated (Figure 2D). With such a low IC_50_ BHT was more effective in preventing ferroptosis when compared with the frequently employed standard ferroptosis-inhibitors ferrostatin-1 and liproxstatin-1 (Figure 2).

### 4.2. The Anti-Ferroptotic GPX4 Is High-Level Expressed in SH-SY5Y Neuroblastoma Cells but ALOX-Isoforms Are Absent

Glutathione peroxidase 4 (GPX4) is a key player in the ferroptosis-signalling cascade [1,2,3]. This enzyme is ubiquitously expressed in most mammalian cells, but the highest expression levels have been reported in the testis. In this organ, the GPX4 mRNA steady-state concentrations are similar to those of GAPDH [60]. To quantify the level of GAPDH expression in SH-SY5Y cells, we carried out qRT-PCR and measured more than 2 × 10^5^ GPX4 mRNA copies per 10^6^ GAPDH mRNA copies (Figure 4B). These data indicate that GPX4 is expressed at rather high levels in SH-SY5Y cells. The major GPX4 isoform in these cells is the cytosolic enzyme since the mRNAs encoding for the mitochondrial and nuclear GPX4 isoforms were detected at much lower steady-state concentrations (Figure 4C,D).

GPX4 activity assays using the lysate supernatant of SH-SY5Y cells as an enzyme source revealed the RSL3-treatment reduced the catalytic activity of GPX4, and this reduction is prevented when the cells were pre-treated with BHT (Figure 6A). The molecular mechanism for the observed GPX4 inhibition by RSL3 in intact SH-SY5Y cells has not been studied in detail. However, the protective effect of BHT suggests the involvement of cellular oxidation reactions, which might lead to the oxidative inactivation of the enzyme. Native GPX4 involves a number of redox-sensitive amino acids including the catalytically active selenocysteine [46,61], and the oxidation of these residues may alter the catalytic activity of the enzyme. Such oxidation reactions may not occur when purified GPX4 is incubated with RSL3 since under these conditions the catalytic activity is not inhibited (Figure 7). Thus, a direct interaction of RLS3 with the GPX4 protein appears unlikely.

Glutathione peroxidase 4 (GPX4) and arachidonic acid lipoxygenases-15 (ALOX15) are antagonising enzymes in the metabolism of hydroperoxy lipids, and they are inversely regulated by cytokines [62]. ALOX15 oxygenates unsaturated ester lipids such as membrane phospholipids [39] and lipoprotein cholesterol esters [40] to their hydroperoxy derivatives, and GPX4 reduces these hydroperoxides to the hydroxy derivatives. Moreover, GPX4 downregulates the catalytic activity of ALOX-isoforms by lowering the intracellular peroxide tone [63]. Since ALOX isoforms have also been implicated in ferroptotic signalling [49,64], we also tested whether ALOX isoforms are expressed in SH-SY5Y cells. The human genome involves six distinct ALOX genes [65]. We worked out qRT-PCR systems including external amplification standards for each ALOX isoform and first tested which of these enzymes are expressed in resting SH-SY5Y cells. Unfortunately, for none of the six different human ALOX isoforms did we obtain reliable amplification signals. These data indicate that ALOX isoforms are not expressed at significant levels in SH-SY5Y cells. To confirm these conclusions for human ALOX15, ALOX15B, ALOX12, and ALOX5, we also performed in vitro activity assays. When incubating the cells in the presence of arachidonic acid we did not detect the formation of specific ALOX products. Even after the stimulation of the cells with RSL3 we neither detected specific amplification signals in qRT-PCR nor the formation of ALOX products in activity assays. Thus, in this cellular model, ALOX isoforms are not needed for RSL3-induced ferroptosis.

### 4.3. RSL3 Inhibits Cellular GPX4 Activity but Does Not Prevent the Catalytic Activity of the Selenocysteine-Containing Recombinant Enzyme

As indicated in Figure 5A, RSL3 inhibits GPX4 activity when SH-SY5Y cells were treated with RSL3. In contrast, when the recombinant selenocysteine-containing enzyme was used, no inhibition was observed (Figure 6). At first glance, these results contradict each other. However, in cellular systems, RSL3 inhibits the catalytic activity of GPX4 but this inhibition may not be caused by direct GPX4-inhibitor interaction. In fact, in a previous study, an adapter protein (14-3-3 epsilon) was identified, which appears to be indispensable for the RSL3-induced inactivation of intracellular GPX4 activity [66]. Overexpression and expression silencing of the 14-3-3 epsilon protein consistently controlled the RSL3-induced inactivation of intracellular GPX4 [66]. The 14-3-3 epsilon protein is encoded by the YWHAE gene, which is located close to the telomere region on the short arm of chromosome 17. On the other hand, RSL3 directly interacts with recombinant human GPX4 (Sec46Cys mutant), and the crystal structure of the enzyme–inhibitor complex indicates that Cys66 may play an important role in this interaction [67]. We found that the selenocysteine-containing human recombinant GPX4 (Figure 5A) is not inhibited by RSL3, and thus the inhibitor may not directly interact with the purified GPX4 protein. This conclusion is consistent with the previous observation that Cys66 is important for enzyme–inhibitor interaction [67] since our enzyme preparation lacks Cys66. The preparation procedure of our selenocysteine-containing GPX4 involved the mutation of all cysteine residues except Cys46, which was expressed in E. coli as Sec using a special type of expression strategy [61].

ML126 is another commercial ferroptosis-inducer [43], and in Figure 3 we show that it induces ferroptosis in SH-SY5Y cells that can be prevented by ferrostatin. When we tested this compound as an inhibitor for recombinant Sec-containing GPX4, we observed 65% inhibition. Thus, ML162 directly interacts with Sec-containing GPX4, and the covalent binding of this compound to Sec has been reported [46]. In other words, no special adapter protein is needed to explain the pro-ferroptotic activity of ML162. However, these data do not exclude the possibility that intracellular adapter proteins may play a role.

### 4.4. BHT Prevents Upregulation of Expression of Patho-Physiologically Relevant Genes in a Rat In Vivo Model of Alzheimer’s Disease

Alzheimer’s disease is the most frequent neurodegenerative disorder [51], and its pathogenesis involves the ferroptotic death of neurons [52]. Here, we observed (Figure 8) that oral the application of BHT prevented the upregulation of the expression of ferroptosis-relevant genes in an in vivo Alzheimer’s disease model. These data suggest that BHT does not only effectively interfere with ferroptotic signalling in cellular in vitro models but that it might also be capable of preventing ferroptosis in vivo. However, our ex vivo expression profiles (Figure 8 and Figure S2) do not prove that BHT protects animals from the development of functional AD symptoms, such as memory defects. More detailed in vivo studies are needed to address this point, and corresponding experiments are currently underway in our group. Moreover, based on our data it was impossible to conclude whether the in vivo effects were related to BHT itself or to BHT metabolites. Moreover, the molecular basis for the observed expression regulations has not been explored. Because of its anti-oxidative properties [17], BHT modifies the intracellular oxidation potential and thus should modify the expression of redox-sensitive genes. However, it remains unclear whether the expression of the quantified genes is redox-sensitive and which transcription factor might be involved.

### 4.5. Degree of Novelty, Advancement of Science, and Limitations of Our Study

It has been reported before that lipophilic anti-oxidants such as vitamin E derivatives or BHT are capable of inhibiting ferroptosis because of their radical-scavenging properties [17,18]. However, the impact of BHT on the RSL3- and ML162-induced ferroptosis of SH-SY5Y neuroblastoma cells has not been reported before. Moreover, we did not only show that BHT inhibits ferroptosis in SH-SY5Y cells but we also explored the molecular basis for its anti-ferroptotic activity. We showed that the degree of oxidation of the cellular membrane lipids is elevated after the induction of ferroptosis (Figure 4) and that BHT suppressed this elevation (Figure 5A). In addition, RSL3 elevated the cellular oxidation potential (Figure 5B), and BHT prevented this effect. Since both the increase in the cellular oxidation potential and the increase in the degree of membrane lipid oxidation are mechanistic elements in ferroptotic signalling, we concluded that BHT inhibits ferroptotic cell death via interfering with these two deleterious processes.

RSL3-induced ferroptotic signalling alters the cellular gene-expression patterns [68,69,70], and the expression alterations of pro- and anti-oxidative enzymes have been implicated. Here, we show that in our cellular ferroptosis model neither the expression of the pro-oxidative ALOX isoforms nor the expression of the anti-oxidative GPX4 was modified. These data prompted the conclusion that ferroptotic signalling in SH-SY5Y cells does not involve ALOX isoforms.

The anti-ferroptotic activity of BHT might be of direct medical interest since the data reported here may qualify BHT as a potential drug for treating ferroptosis-related diseases. This may be the case for different neurodegenerative diseases, for which ferroptosis has been suggested as a relevant patho-physiological mechanism, but also for diseases of the cardiovascular, urinary, reproductive, and the digestive systems [3]. The data presented in Figure 8 indicate that oral BHT administration normalized the cerebral expression of ferroptosis-related genes. Whether these observed expression regulations are a functional consequence of ferroptosis inhibition can hardly be judged at the moment, but work is in progress in our laboratory to address this point.

Ferroptosis is not always a deleterious process since it also constitutes a physiological defence mechanism in tumour biology. Ferroptosis kills cancer cells, and thus it prevents tumour growth and metastasis [1,2,3]. In other words, the systemic application of BHT in humans might slow down the development of neurodegenerative diseases but may also facilitate the growth of tumours. In fact, the pro-carcinogenic effects of BHT, which have previously been described to occur at high BHT concentrations [23,24,25], might be related to the inhibition of tumour-cell ferroptosis. Thus, before the clinical use of BHT as a ferroptosis-inhibitor in neurodegenerative diseases can be recommended, more detailed mechanistic studies on the possible adverse effects of this compound are needed.

## 5. Conclusions

Butylated hydroxytoluene (BHT) is frequently used as an anti-oxidative preservative in oil chemistry and cosmetics but owing to the adverse health effects its use in food chemistry it has not been recommended any more. Here, we report that BHT effectively prevents RSL3- and ML162-induced ferroptotic cell death in a human neuroblastoma cell line by inhibiting the ferroptosis-related oxidation of membrane lipids. Its anti-ferroptotic effect was confirmed in an in vivo rat model of Alzheimer’s disease. Taken together, our results suggest that BHT might exhibit neuroprotective effects preventing ferroptosis-induced neuronal cell death. Eventually, Alzheimer’s disease patients might benefit from BHT treatment.

## Data Availability

The original experimental raw data can be obtained upon request from P.F., S.A. and H.K.

## Supplementary Materials

The supplementary materials can be found at https://www.mdpi.com/article/10.3390/antiox13020242/s1. Figure S1. RP-HPLC analysis of the oxidation degree of SH-SY5Y membrane lipids; Figure S2. Streptozotocin treatment induced the expression of the APP gene but pre-treatment of the animals with BHT prevented this effect.

## References

[1] Stockwell B.R. (2022). Ferroptosis turns 10: Emerging mechanisms, physiological functions, and therapeutic applications. Cell.

[2] Jiang X., Stockwell B.R., Conrad M. (2021). Ferroptosis: Mechanisms, biology and role in disease. Nat. Rev. Mol. Cell Biol..

[3] Tang D., Chen X., Kang R., Kroemer G. (2021). Ferroptosis: Molecular mechanisms and health implications. Cell Res..

[4] Dixon S.J., Lemberg K.M., Lamprecht M.R., Skouta R., Zaitsev E.M., Gleason C.E., Patel D.N., Bauer A.J., Cantley A.M., Yang W.S. (2012). Ferroptosis: An iron-dependent form of nonapoptotic cell death. Cell.

[5] Liu J., Kang R., Tang D. (2022). Signaling pathways and defense mechanisms of ferroptosis. FEBS J..

[6] Rochette L., Dogon G., Rigal E., Zeller M., Cottin Y., Vergely C. (2022). Lipid Peroxidation and Iron Metabolism: Two Corner Stones in the Homeostasis Control of Ferroptosis. Int. J. Mol. Sci..

[7] Chen X., Li J., Kang R., Klionsky D.J., Tang D. (2021). Ferroptosis: Machinery and regulation. Autophagy.

[8] David S., Jhelum P., Ryan F., Jeong S.Y., Kroner A. (2022). Dysregulation of Iron Homeostasis in the Central Nervous System and the Role of Ferroptosis in Neurodegenerative Disorders. Antioxid. Redox Signal..

[9] Papanikolaou G., Pantopoulos K. (2005). Iron metabolism and toxicity. Toxicol. Appl. Pharmacol..

[10] Venkataramani V. (2021). Iron Homeostasis and Metabolism: Two Sides of a Coin. Adv. Exp. Med. Biol..

[11] Griffiths H.R., Gao D., Pararasa C. (2017). Redox regulation in metabolic programming and inflammation. Redox Biol..

[12] Lee B.W.L., Ghode P., Ong D.S.T. (2019). Redox regulation of cell state and fate. Redox Biol..

[13] Sies H. (2015). Oxidative stress: A concept in redox biology and medicine. Redox Biol..

[14] Forcina G.C., Dixon S.J. (2019). GPX4 at the Crossroads of Lipid Homeostasis and Ferroptosis. Proteomics.

[15] Zheng J., Conrad M. (2020). The Metabolic Underpinnings of Ferroptosis. Cell Metab..

[16] Zhang B., Pan C., Feng C., Yan C., Yu Y., Chen Z., Guo C., Wang X. (2022). Role of mitochondrial reactive oxygen species in homeostasis regulation. Redox Rep..

[17] Yehye W.A., Rahman N.A., Ariffin A., Abd Hamid S.B., Alhadi A.A., Kadir F.A., Yaeghoobi M. (2015). Understanding the chemistry behind the antioxidant activities of butylated hydroxytoluene (BHT): A review. Eur. J. Med. Chem..

[18] Burton G.W., Doba T., Gabe E.J., Hughes L., Lee F.L., Prasad L., Ungold K.U. (1985). Autoxidation of Biological Molecules. 4. Maximizing the Antioxidant Activity of Phenols. J. Am. Chem. Soc..

[19] Snipes W., Person S., Keith A., Cupp J. (1975). Butylated hydroxytoluene inactivated lipid-containing viruses. Science.

[20] Yamamoto K., Tajima K., Mizutani T. (1980). The acute toxicity of butylated hydroxytoluene and its metabolites in mice. Toxicol. Lett..

[21] Lanigan R.S., Yamarik T.A. (2002). Final report on the safety assessment of BHT(1). Int. J. Toxicol..

[22] Williams G.M., Iatropoulos M.J., Whysner J. (1999). Safety assessment of butylated hydroxyanisole and butylated hydroxytoluene as antioxidant food additives. Food Chem. Toxicol..

[23] Bomhard E.M., Bremmer J.N., Herbold B.A. (1992). Review of the mutagenicity/genotoxicity of butylated hydroxytoluene. Mutat. Res..

[24] Botterweck A.A., Verhagen H., Goldbohm R.A., Kleinjans J., van den Brandt P.A. (2000). Intake of butylated hydroxyanisole and butylated hydroxytoluene and stomach cancer risk: Results from analyses in the Netherlands Cohort Study. Food Chem. Toxicol..

[25] Thompson J.A., Bolton J.L., Malkinson A.M. (1991). Relationship between the Metabolism of Butylated Hydroxytoluene (Bht) and Lung-Tumor Promotion in Mice. Exp. Lung Res..

[26] Bligh E.G., Dyer W.J. (1959). A Rapid Method of Total Lipid Extraction and Purification. Can. J. Biochem. Phys..

[27] Huang L.S., Kang J.S., Kim M.R., Sok D.E. (2008). Oxygenation of arachidonoyl lysophospholipids by lipoxygenases from soybean, porcine leukocyte, or rabbit reticulocyte. J. Agric. Food Chem..

[28] Akhtar A., Gupta S.M., Dwivedi S., Kumar D., Shaikh M.F., Negi A. (2022). Preclinical Models for Alzheimer’s Disease: Past, Present, and Future Approaches. ACS Omega.

[29] Silva S.S.L., Tureck L.V.V., Souza L.C., Mello-Hortega J.V.V., Piumbini A.L., Teixeira M.D., Furtado-Alle L., Vital M.A.B.F., Souza R.L.R. (2023). Animal model of Alzheimer’s disease induced by streptozotocin: New insights about cholinergic pathway. Brain Res..

[30] Duan L., Qian X., Wang Q., Huang L., Ge S. (2022). Experimental Periodontitis Deteriorates Cognitive Function and Impairs Insulin Signaling in a Streptozotocin-Induced Alzheimer’s Disease Rat Model. J. Alzheimer’s Dis..

[31] Chen Z.Y., Zhang Y. (2022). Animal models of Alzheimer’s disease: Applications, evaluation, and perspectives. Zool. Res..

[32] Scarpellini C., Klejborowska G., Lanthier C., Hassannia B., Vanden Berghe T., Augustyns K. (2023). Beyond ferrostatin-1: A comprehensive review of ferroptosis inhibitors. Trends Pharmacol. Sci..

[33] Chen H., Qi Q., Wu N., Wang Y., Feng Q., Jin R., Jiang L. (2022). Aspirin promotes RSL3-induced ferroptosis by suppressing mTOR/SREBP-1/SCD1-mediated lipogenesis in PIK3CA-mutant colorectal cancer. Redox Biol..

[34] Kawasaki N.K., Suhara T., Komai K., Shimada B.K., Yorichika N., Kobayashi M., Baba Y., Higa J.K., Matsui T. (2023). The role of ferroptosis in cell-to-cell propagation of cell death initiated from focal injury in cardiomyocytes. Life Sci..

[35] Shintoku R., Takigawa Y., Yamada K., Kubota C., Yoshimoto Y., Takeuchi T., Koshiishi I., Torii S. (2017). Lipoxygenase-mediated generation of lipid peroxides enhances ferroptosis induced by erastin and RSL3. Cancer Sci..

[36] Chen T.T., Leng J.F., Tan J., Zhao Y.J., Xie S.S., Zhao S.F., Yan X., Zhu L., Luo J., Kong L. (2023). Discovery of Novel Potent Covalent Glutathione Peroxidase 4 Inhibitors as Highly Selective Ferroptosis Inducers for the Treatment of Triple-Negative Breast Cancer. J. Med. Chem..

[37] van Leyen K., Duvoisin R.M., Engelhardt H., Wiedmann M. (1998). A function for lipoxygenase in programmed organelle degradation. Nature.

[38] Banthiya S., Pekarova M., Kuhn H., Heydeck D. (2015). Secreted lipoxygenase from Pseudomonas aeruginosa exhibits biomembrane oxygenase activity and induces hemolysis in human red blood cells. Arch. Biochem. Biophys..

[39] Kuhn H., Belkner J., Wiesner R., Brash A.R. (1990). Oxygenation of biological membranes by the pure reticulocyte lipoxygenase. J. Biol. Chem..

[40] Belkner J., Wiesner R., Rathman J., Barnett J., Sigal E., Kuhn H. (1993). Oxygenation of lipoproteins by mammalian lipoxygenases. Eur. J. Biochem..

[41] Escada-Rebelo S., Ramalho-Santos J. (2023). Oxidative and Nitrosative Stress Detection in Human Sperm Using Fluorescent Probes. Methods Mol. Biol..

[42] Ursini F., Maiorino M., Roveri A. (1997). Phospholipid hydroperoxide glutathione peroxidase (PHGPx): More than an antioxidant enzyme?. Biomed. Environ. Sci..

[43] Cheff D.M., Huang C., Scholzen K.C., Gencheva R., Ronzetti M.H., Cheng Q., Hall M.D., Arner E.S.J. (2023). The ferroptosis inducing compounds RSL3 and ML162 are not direct inhibitors of GPX4 but of TXNRD1. Redox Biol..

[44] Sui X.B., Zhang R.N., Liu S.P., Duan T., Zhai L.J., Zhang M.M., Han X., Xiang Y., Huang X., Lin H. (2018). RSL3 Drives Ferroptosis Through GPX4 Inactivation and ROS Production in Colorectal Cancer. Front. Pharmacol..

[45] Sekhar K.R., Hanna D.N., Cyr S., Baechle J.J., Kuravi S., Balusu R., Rathmel K., Baregamian N. (2022). Glutathione peroxidase 4 inhibition induces ferroptosis and mTOR pathway suppression in thyroid cancer. Sci. Rep..

[46] Moosmayer D., Hilpmann A., Hoffmann J., Schnirch L., Zimmermann K., Badock V., Furst L., Eaton J.K., Viswanathan V.S., Schreiber S.L. (2021). Crystal structures of the selenoprotein glutathione peroxidase 4 in its apo form and in complex with the covalently bound inhibitor ML162. Acta Crystallogr. D Struct. Biol..

[47] Probst L., Dachert J., Schenk B., Fulda S. (2017). Lipoxygenase inhibitors protect acute lymphoblastic leukemia cells from ferroptotic cell death. Biochem. Pharmacol..

[48] Liu Y., Wang W., Li Y., Xiao Y., Cheng J., Jia J. (2015). The 5-Lipoxygenase Inhibitor Zileuton Confers Neuroprotection against Glutamate Oxidative Damage by Inhibiting Ferroptosis. Biol. Pharm. Bull..

[49] Wenzel S.E., Tyurina Y.Y., Zhao J., St Croix C.M., Dar H.H., Mao G., Tyurin V.A., Anthonymuthu T.S., Kapralov A.A., Amoscato A.A. (2017). PEBP1 Wardens Ferroptosis by Enabling Lipoxygenase Generation of Lipid Death Signals. Cell.

[50] Khan S., Barve K.H., Kumar M.S. (2020). Recent Advancements in Pathogenesis, Diagnostics and Treatment of Alzheimer’s Disease. Curr. Neuropharmacol..

[51] Zhang X.X., Tian Y., Wang Z.T., Ma Y.H., Tan L., Yu J.T. (2021). The Epidemiology of Alzheimer’s Disease Modifiable Risk Factors and Prevention. J. Prev. Alzheimer’s Dis..

[52] Ma H., Dong Y., Chu Y., Guo Y., Li L. (2022). The mechanisms of ferroptosis and its role in alzheimer’s disease. Front. Mol. Biosci..

[53] Yan N., Zhang J. (2019). Iron Metabolism, Ferroptosis, and the Links With Alzheimer’s Disease. Front. Neurosci..

[54] Jafarzadeh G., Shakerian S., Farbood Y., Ghanbarzadeh M. (2021). Effects of Eight Weeks of Resistance Exercises on Neurotrophins and Trk Receptors in Alzheimer Model Male Wistar Rats. Basic. Clin. Neurosci..

[55] Plays M., Muller S., Rodriguez R. (2021). Chemistry and biology of ferritin. Metallomics.

[56] Park E., Chung S.W. (2019). ROS-mediated autophagy increases intracellular iron levels and ferroptosis by ferritin and transferrin receptor regulation. Cell Death Dis..

[57] Doll S., Proneth B., Tyurina Y.Y., Panzilius E., Kobayashi S., Ingold I., Irmler M., Beckers J., Aichler M., Walch A. (2017). ACSL4 dictates ferroptosis sensitivity by shaping cellular lipid composition. Nat. Chem. Biol..

[58] Lane C.A., Hardy J., Schott J.M. (2018). Alzheimer’s disease. Eur. J. Neurol..

[59] Nieva-Echevarria B., Manzanos M.J., Goicoechea E., Guillen M.D. (2015). 2,6-Di-Tert-Butyl-Hydroxytoluene and Its Metabolites in Foods. Compr. Rev. Food Sci. Food Saf..

[60] Borchert A., Savaskan N.E., Kuhn H. (2003). Regulation of expression of the phospholipid hydroperoxide/sperm nucleus glutathione peroxidase gene. Tissue-specific expression pattern and identification of functional cis- and trans-regulatory elements. J. Biol. Chem..

[61] Borchert A., Kalms J., Roth S.R., Rademacher M., Schmidt A., Holzhutter H.G., Kuhn H., Scheerer P. (2018). Crystal structure and functional characterization of selenocysteine-containing glutathione peroxidase 4 suggests an alternative mechanism of peroxide reduction. Biochim. Biophys. Acta Mol. Cell Biol. Lipids.

[62] Schnurr K., Borchert A., Kuhn H. (1999). Inverse regulation of lipid-peroxidizing and hydroperoxyl lipid-reducing enzymes by interleukins 4 and 13. FASEB J..

[63] Weitzel F., Wendel A. (1993). Selenoenzymes regulate the activity of leukocyte 5-lipoxygenase via the peroxide tone. J. Biol. Chem..

[64] Kagan V.E., Mao G., Qu F., Angeli J.P., Doll S., Croix C.S., Hussain Dar H., Liu B., Tyurin V.A., Ritov V.B. (2017). Oxidized arachidonic and adrenic PEs navigate cells to ferroptosis. Nat. Chem. Biol..

[65] Funk C.D., Chen X.S., Johnson E.N., Zhao L. (2002). Genes and their targeted disruption. Prostaglandins Other Lipid Mediat..

[66] Vuckovic A.M., Bosello Travain V., Bordin L., Cozza G., Miotto G., Rossetto M., Toppo S., Venerando R., Zaccarin M., Maiorino M. (2020). Inactivation of the glutathione peroxidase GPx4 by the ferroptosis-inducing molecule RSL3 requires the adaptor protein 14-3-3epsilon. FEBS Lett..

[67] Liu H., Forouhar F., Lin A.J., Wang Q., Polychronidou V., Soni R.K., Xia X., Stockwell B.R. (2022). Small-molecule allosteric inhibitors of GPX4. Cell Chem. Biol..

[68] Gatt A., Lee H., Williams G., Thuret S., Ballard C. (2019). Expression of neurogenic markers in Alzheimer’s disease: A systematic review and metatranscriptional analysis. Neurobiol. Aging.

[69] Sharma V.K., Mehta V., Singh T.G. (2020). Alzheimer’s Disorder: Epigenetic Connection and Associated Risk Factors. Curr. Neuropharmacol..

[70] Li D., Yang H.X., Lyu M., Zhou L.H., Zhang Y., Kang C.S., Wang J., Wang Y. (2023). Association between behavioural risks and Alzheimer’s disease: Elucidated with an integrated analysis of gene expression patterns and molecular mechanisms. Neurosci. Biobehav. R..

