# Supplementary material for: Butylated Hydroxytoluene (BHT) Protects SH-SY5Y Neuroblastoma Cells from Ferroptotic Cell Death: Insights from In Vitro and In Vivo Studies
Source: Antioxidants (Basel). 2024 Feb 17;13(2):242. doi: 10.3390/antiox13020242 (PMC10886092; doi:10.3390/antiox13020242)
Supplement: Supplementary file 1 [file antioxidants-13-00242-s001.zip › antioxidants-2789511-supplementary.pdf]

antioxidants

Online supplement for the paper

Butylated Hydroxytoluene (BHT) Protects SH-SY5Y Neuroblastoma Cells from Ferroptotic Cell Death: Insights from In Vitro and In Vivo Studies

Parisa Faraji [1,2], Astrid Borchert [1], Shahin Ahmadian [2,*] and Hartmut Kuhn [1,*]

[1] Department of Biochemistry, Charité—Universitätsmedizin Berlin, Corporate member of Freie Universität Berlin and Humboldt Universität zu Berlin, Charitéplatz 1, 10117 Berlin, Germany; parisa.faraji@charite.de (P.F.); astrid.borchert@charite.de (A.B.)
[2] Institute of Biochemistry and Biophysics, University of Tehran,1417614335, Tehran, Iran
* Correspondence: sh.ahmadian@ut.ac.ir (S.A.); hartmut.kuehn@charite.de (H.K.); Tel.: +49-30-450-528-040

1. Supplemental HPLC chromatorams of hydrolyzed lipid extracts of SH-SY5Y cells

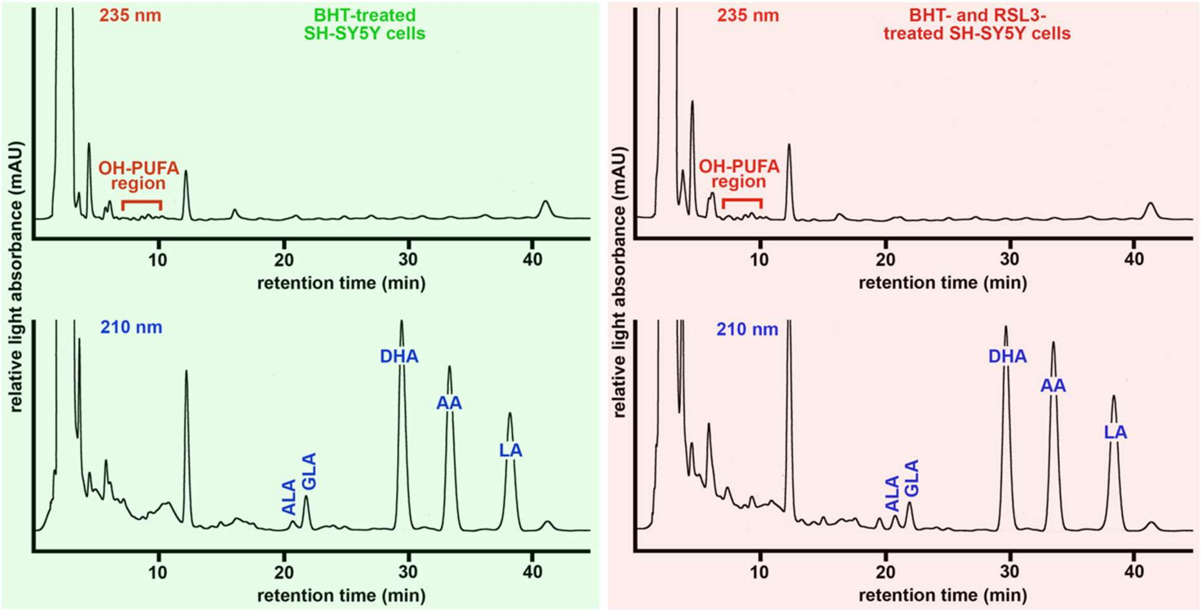

Figure S1. RP-HPLC analysis of the oxidation degree of SH-SY5Y membrane lipids. SH-SY5Y cells were grown to near confluency in 10 cm Petri dishes and and pre-incubated for 2 h with 1 µM of BHT. Then ferroptosis was induced by the addition of 10 µM RSL3 in the RSL3 group. For the BHT group an appropriate amount of PBS was added. After a two-hour incubation period the cells were harvested, washed and samples were further worked-up and analyzed as described in of legend to Figure 4. A) Analysis of hydroxy-PUFA of confluent SH-SY5Y cells cultured in the presence of 1 µM BHT. B) Analysis of non-oxidized PUFAs of SH-SY5Y cells cultured in the absence of 10 ¥µM RSL3 and 1 µM BHT.

2. Expression of the *APP* gene in the hypothalamus of streptozotocin-treated rats (AD group), SHAM operated animals (AD group) and streptozotocin-treated rats that were previously pre-treated with BHT.

To explore the alterations in the gene expression patterns that were induced by streptozotocin-treatment of the rats we quantified the expression of AD related genes (Fth1, Acsl4, Alox15) in the hippocampus (Figure 8 of main paper). Since the amyloid precursor protein (APP) is of major patho-physiological relevance for AD we also quantified the tissue concentrations of the APP mRNA in the hippocampus. Here we obtained specific amplification signals and these signals were more intense after induction of the AD symptoms. Although the difference between the two groups did not reach the level of statistical significance. Most interestingly, BHT treatment prevented the increase in APP expression and we observed a significant difference ($p < 0.05$) between the AD-rats (no BHT administration) and the HBT-treated AD animals **(see Figure S2)**.

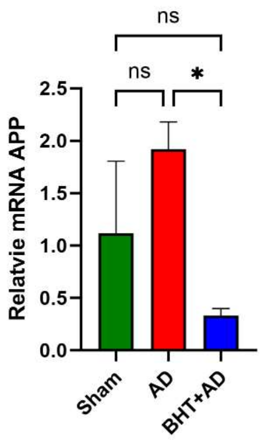

Figure S2. Streptozotocin treatment induced the expression of the APP gene but pre-treatment of the animals with BHT prevented this effect. Rats were orally pre-treated with BHT at a dose of 120 mg/kg body weight for 4 days and then the formation of AD-related symptoms was induced by bilateral intracerebroventricular injections of streptozotocin (3 mg/kg on both sides). Hippocampal tissue was prepared, total RNA was extracted and semi-quantitative RT-PCR was carried out using an APP specific primer combination AAA ATG AAG TTG AGC CTG TCG (forward) and GTT TGT CAA CCC AGA CCC TG (reverse). RT-PCR was carried out as described in Section 2.7.
